# Clinically helpful rickettsial disease diagnostic IgG titers in relation to duration of illness in an endemic setting in Sri Lanka

**DOI:** 10.1186/1756-0500-5-662

**Published:** 2012-11-30

**Authors:** Ranjan Premaratna, Sanjaya Weerasinghe, Amanda Ranaweera, TGA Nilmini Chandrasena, Narasinghe W Bandara, Gregory A Dasch, H Janaka de Silva

**Affiliations:** 1 Department of Medicine, Faculty of Medicine, University of Kelaniya, Colombo, Sri Lanka; 2 Department of Parasitology, Faculty of Medicine, University of Kelaniya, Colombo, Sri Lanka; 3 Department of Microbiology, Faculty of Medicine, University of Kelaniya, Colombo, Sri Lanka; 4 Rickettsial Zoonoses Branch, CDC, Atlanta, GA, USA

**Keywords:** Diagnosis Rickettsioses, IFA-IgG, Endemic, Sri Lanka, Orientia tsutsugamushi, Spotted fever

## Abstract

**Background:**

Although an initial IFA-IgG titer greater or equal to 1/64 or 1/128 is considered positive in presumptive diagnosis, in clinical practice in an endemic setting for rickettsioses in Sri Lanka, some patients with IFA-IgG titer of 1/128 for either spotted fever group (SFG) or scrub typhus (ST) did not respond to treatment.

**Findings:**

To determine a clinically helpful diagnostic algorithm, IFA-IgG results of serologically confirmed treatment responders were analyzed in relation to duration of illness at sampling. Of 146 suspected SFG, 3 responders of 25 patients had titers ≤1/128 with < 7 days of illness while all 9 with titers ≥1/256 responded (false negative with 1/256 cutoff was 12%, false positive was 0%). For illness > 7 days, the false negative and positive rates were 4.3% (3/59) and 11.3% (6/53). Of 115 suspected ST, false negative and positive rates with ≥1/256 cutoff at <7 days of illness were 14.2% (2/14) and 0% (0/8) respectively while > 7 days, false negative and positive rates were 2% (1/51) and 0% (0/42).

**Conclusions:**

For clinical decision making, duration of illness at sampling is important in interpreting serology results in an endemic setting. If sample is obtained ≤7 day of illness, an IgG titer of ≤1/128 requires a follow up sample in the diagnosis and > 7 days of illness, a single ≥1/256 titer is diagnostic for all ST and 90% of SFG.

## Findings

### Introduction

Rickettsioses are prevalent in many geographical areas of the world. Man is an accidental host and when infected, results in a diverse clinical picture. The presence of an eschar or a discrete maculo-papular rash helps in the diagnosis [1]. Most patients have other varying clinical manifestations which are nonspecific [2]. Presentation as pyrexia of unknown origin is a fairly common scenario of rickettsioses in an endemic setting, in addition to extended morbidity, severe complications and mortality [3].

In most endemic areas the diagnosis of rickettsioses is mostly on clinical grounds as rickettsial disease confirmatory facilities are mostly available in reference laboratories. Despite assay misinterpretations, non-specific cross-reactions, and delayed antibody responses in some patients, the mainstay of laboratory diagnosis is based on detection by immuno-fluorescent assays (IFA) of human IgG or IgM responses against rickettsial antigens [4-6]. Although PCR based diagnostic methods are available in most reference laboratories, its availability as a reliable and rapid clinical diagnostic tool is very limited [6].

As for many other illnesses where the laboratory diagnosis is based on serology, a fourfold increase or a decline in antibody titers is required for confirmation of the illness. Because the convalescent serum has not yet been collected, clinical management is based on a presumptive diagnosis or empirical basis made on disease-compatible clinical features together with a single IFA-IgM titer or an IFA- IgG titer greater than a set cutoff level for the geographical region [6]. However, detection of IgM in the diagnosis of acute rickettsioses is unreliable due to its cross reactivity with other bacterial antigens [6]. The current presumptive diagnostic titer for IFA-IgG in a single acute serum sample is set at ≥ 1/64 or ≥1/128 by different laboratories based on the background sero-prevalence of rickettsioses in a given geographical location [6,7].

In Sri Lanka, rickettsioses comprise a significant portion of acute febrile illness and pyrexia of uncertain origin and it is the 5^th^ most common infectious disease. Until 2008, the diagnosis of rickettsioses within the country was based only on clinical features, or inferred by clinical response to anti-rickettsial antibiotics or the non-specific Weil Felix test, which has many limitations for scrub typhus. In June 2008, rickettsial disease diagnostics based on IFA was established in collaboration with CDC Atlanta, Georgia, USA. Since then, it has become increasingly apparent that many geographic locations of the island have a high background sero-prevalence of IgG antibodies against *O. tsutsugamushi* the organism responsible for scrub typhus (ST) and against spotted fever group (SFG) rickettsioses, making it more difficult to interpret a single IFA-IgG titer in the diagnosis of acute rickettsioses in patients with acute febrile illness (unpublished data). Furthermore, as most requests for rickettsial disease diagnostics are done at a late stage of clinical illness, these factors cause difficulties in interpreting initial IgG titers which are below 128. Therefore, we felt there was a need for clinically useful diagnostic algorithms for interpreting IgG titers based on the duration of clinical illness at presentation of the patient in different endemic settings.

### Objectives

In this study, we aimed to identify a clinically useful diagnostic algorithm for interpreting IgG titers taking into account the duration of clinical illness at presentation when the first serum sample was collected.

### Methods

A database was maintained prospectively at the Rickettsial Disease Diagnostic and Research Laboratory (RDDRL), Faculty of Medicine, University of Kelaniya, Sri Lanka from its inception in June 2008. The data included in the database consisted of clinical details and laboratory investigation results which were provided in a detailed structured request form that should accompany the appropriate blood or serum samples at the time the rickettsial diseases diagnostic tests were requested. The database was updated with the rickettsial diseases IFA-IgG titres and also with the details of clinical response with appropriate anti-rickettsial antibiotics. The database of RDDRL, which contains IgG results for clinical cases of suspected rickettsioses (potential cases) on admission based on clinical features and basic investigations was then retrospectively analyzed in relation to the known duration of illness at the time of sampling and whether the patients responded favorably to anti-rickettsial antibiotics such as doxycycline or azithromycin. Out of which, those who were confirmed having rickettsioses either by seroconversion on a follow up sample after 2–3 weeks or who had initial very high titres (>1/512) and responded to anti-rickettsial antibiotics were selected. Presently we consider rising IFA-IgG titres as gold standard in the diagnosis of acute rickettsioses as we do not have PCR based rickettsial disease diagnostics. Based on CDC recommendations, an initial IFA-IgG titre of 1/128 is currently used in the presumptive diagnosis of acute rickettsioses on acute serum samples obtained at admission in clinically suspected cases. A respondant was defined, when the fever subsided within 48–72 hours of initiating anti-rickettsial antibiotics. In the RDDRL, IFA assays were carried out using rickettsial antigens grown in culture: *Rickettsia conorii* (Malish) and *Orientia tsutsugamushi* (Karp). Antibodies were detected using fluorescein conjugated goat anti-human IgG(γ) or IgM(μ) (KPL, Inc., Gaithersburg, MD, USA). Ethical clearance to publish these data without revealing patient personal details of identities was obtained from the Ethics Review Committee, Faculty of Medicine, University of Kelaniya, Sri Lanka.

### Results

Out of 478 samples that had been analyzed for rickettsioses by November 2010, the date of collection in relation to illness and follow up serology was known in 261 (Table 1). Fifty six had been sent on or before the 7^th^ day of illness with a mean of 5 days, and 205 were sent after the 7^th^ day with a mean of 19 days. The number positive at different titers on initial serum sampling, (reciprocal of last positive endpoint dilution) their sero-conversion and response to treatment are given in the table.

**Table 1:** Admission IFA-IgG titres confirming SFG and ST in relation to duration of illness

**Disease**	**Total**	**Duration of illness when serum was obtained ≤ 7 days**					
		**≤1/128**	**Sero-converted**	**Responded**	**≥1/256**	**Sero-converted (High initial titres)**	**Responded**
SFG	34	25	3	3	9	7 (2)	All (9)
ST	22	14	2	2	8	5 (3)	All (8)
Disease	Total	Duration of illness when serum was obtained > 7 days					
		≤1/128	Sero-converted	Responded	≥1/256	Sero-converted (High initial titres)	Responded
SFG	112	59	3	3	53	21 (26)	47
ST	93	51	1	1	42	16 (26)	All (42)

Of the 146 suspected SFG infections, only 3 responders of 25 patients had titers ≤ 1/128 with less than 7 days of illness while all 9 with titers ≥ 1/256 responded (false negative with 1/256 cutoff was 12%, false positive was 0%). For illness that was greater than 7 days, the false negative and positive rates at ≥ 1/256 were 5% (3/59) and 11.3% (6/53). For the 115 suspected ST infections false negative and positive rates with ≥ 1/256 cutoff at less than 7 days of illness were (2/14) and 0% (0/8) respectively while with illness greater than 7 days, false negative and positive rates were 2% (1/51) and 0% (0/42).

### Discussion

This study helped us to formulate a clinically useful diagnostic algorithm considering the duration of clinical illness at the time when serum samples are obtained for IgG diagnosis of rickettsioses in an endemic setting. There are two groups of serum samples that require mention about its meaning concerning their diagnostic values: i. titer ≥ 1/256, ≤ 7days of illness, ii. Titer ≤1/128, > 7 days of illness. When the sample is obtained > 7 days of illness, a single ≥ 1/256 titer is diagnostic for all scrub typhus (ST) infections and 90% of SFG infections. However, for either SFG or ST, if the sample is obtained ≤ 7 day of illness, an IgG titer of ≤ 1/128 requires a follow up sample in the diagnosis. Most patients who had a 1/128 or 1/64 titer (the cutoff which is recommended by the CDC in the presumptive diagnosis of acute rickettsioses) in a sample obtained after the 7^th^ day of illness had no clinical rickettsioses. Our findings are in keeping with a higher diagnostic cutoff titers recommended by some commercially available rapid diagnostic test kits [8].

Furthermore, it is important to identify the species causing the rickettsioses that are prevalent in a given geographical location because it is known that some rickettsial species are known to induce delayed immunological responses and therefore, a delayed rise in IgM and IgG titres [6,8,9]. One drawback in our results is that we are still unaware of the spectrum of SFG and ST rickettsial organisms causing disease within our geography and whether higher titers against autochthonous SFG or ST antigens might be obtained than with *R. conorii* or *Orientia tsutsugamushi* Karp. Therefore, it is recommended that while initial higher IgG titers against rickettsioses seem to be more accurate in the diagnosis of acute rickettsioses, further studies are needed in order to identify the spectrum of rickettsioses in the country. Consequently, another study to look at concomitant changes in IFA-IgM titers may help to clarify uncertainties of mixed IFA-IgG titers against SFG, murine typhus, or ST in the diagnosis of acute rickettsioses in the country. However, cost is the major drawback in additional evaluation of IgM titers in the diagnosis of rickettsioses in our setting.

## Competing interests

The authors declare that they have no competing interests.

## Authors’ contributions

RP: Designing the study, Analyzing data, writing up the paper. GAD, HjdeS: Analyzing data, writing up the paper. SW, AR: Analyzing data, TGANC, NWB: maintaining database, analyzing data. All authors read and approved the final manuscript.

## Disclosure

This paper was presented at the 6^th^ International Meeting on Rickettsiae and Rickettsial Diseases, Heraklion, Crete, Greece, 5–7 June, 2011; paper O015. The findings and conclusions in this report are those of the authors and do not necessarily represent the views of the United States Department of Health and Human Services or the Centers for Disease Control and Prevention.
